# Phylogenetic group- and species-specific oligonucleotide probes for single-cell detection of lactic acid bacteria in oral biofilms

**DOI:** 10.1186/1471-2180-11-14

**Published:** 2011-01-19

**Authors:** Beatrice Quevedo, Elin Giertsen, Vincent Zijnge, Helga Lüthi-Schaller, Bernhard Guggenheim, Thomas Thurnheer, Rudolf Gmür

**Affiliations:** 1 Institute of Oral Biology, Section of Oral Microbiology and General Immunology, University of Zürich Plattenstrasse 11, 8032 Zürich, Switzerland; 2 Institute of Clinical Dentistry, Department of Cariology and Gerodontology, Faculty of Dentistry, University of Oslo, Oslo, Norway; 3 Center for Dentistry and Oral Hygiene and Department of Medical Microbiology, University of Groningen, Groningen, The Netherlands

## Abstract

**Background:**

The purpose of this study was to design and evaluate fluorescent *in situ *hybridization (FISH) probes for the single-cell detection and enumeration of lactic acid bacteria, in particular organisms belonging to the major phylogenetic groups and species of oral lactobacilli and to *Abiotrophia/Granulicatella*.

**Results:**

As lactobacilli are known for notorious resistance to probe penetration, probe-specific assay protocols were experimentally developed to provide maximum cell wall permeability, probe accessibility, hybridization stringency, and fluorescence intensity. The new assays were then applied in a pilot study to three biofilm samples harvested from variably demineralized bovine enamel discs that had been carried *in situ *for 10 days by different volunteers. Best probe penetration and fluorescent labeling of reference strains were obtained after combined lysozyme and achromopeptidase treatment followed by exposure to lipase. Hybridization stringency had to be established strictly for each probe. Thereafter all probes showed the expected specificity with reference strains and labeled the anticipated morphotypes in dental plaques. Applied to *in situ *grown biofilms the set of probes detected only *Lactobacillus fermentum *and bacteria of the *Lactobacillus casei *group. The most cariogenic biofilm contained two orders of magnitude higher *L. fermentum *cell numbers than the other biofilms. *Abiotrophia/Granulicatella *and streptococci from the mitis group were found in all samples at high levels, whereas *Streptococcus mutans *was detected in only one sample in very low numbers.

**Conclusions:**

Application of these new group- and species-specific FISH probes to oral biofilm-forming lactic acid bacteria will allow a clearer understanding of the supragingival biome, its spatial architecture and of structure-function relationships implicated during plaque homeostasis and caries development. The probes should prove of value far beyond the field of oral microbiology, as many of them detect non-oral species and phylogenetic groups of importance in a variety of medical conditions and the food industry.

## Background

It is estimated that more than 10^10 ^bacteria per gram of dental plaque colonize the human oral cavity. More than half of them still remain uncultivable. Their existence is only known because a fingerprint in form of a sequence from a gene fragment, most often from 16S rDNA, could be traced in a clinical sample [1]. All oral microorganisms form biofilms on surfaces such as the oral mucosa, the tongue, or the surface of the teeth. Many supragingivally predominant bacteria belong to the *Firmicutes *phylum (Gram-positive rods and cocci of low G+C content) with the lactic acid producing bacteria (LAB) as the largest and clinically important subgroup [2,3]. Comprising streptococci, lactobacilli, and *Granulicatella/Abiotrophia *species (formerly described as nutritionally variant streptococci), LAB are main constituents of the commensal microbiota of the human oral cavity, but form also part of the biofilms colonizing the upper respiratory, intestinal and urinary tracts. In the oral cavity, they are thought to play major roles in dental plaque formation and oral biofilm homeostasis. However, under conditions of prolonged shifts of biofilm composition, LAB may induce dental caries through excessive lactic acid formation [4], and upon penetration into the blood stream LAB may cause in susceptible individuals a variety of life-threatening conditions such as endocarditis, septicemia, or meningitis [5,6].

*In situ *techniques that allow monitoring individual cells and cell populations within biofilms are important tools to investigate natural biofilm ecologies [7,8]. However, few probes for the detection and quantification by fluorescent *in situ *hybridization (FISH) of oral LAB species have been described so far [9,10]. Here we report the design, characterization and pilot evaluation of probes recognizing major phylogenetic clusters or species of oral lactobacilli, the *Abiotrophia/Granulicatella *group, and a few taxa of oral streptococci. Applied for validation to *in situ *formed supragingival biofilms, the probes detected high levels of both mitis group streptococci and *Abiotrophia/Granulicatella *species, and identified strains of *Lactobacillus fermentum *and the *Lactobacillus casei *group.

(The study is part of the requirements for BQ's Doctor degree of Dental Medicine.)

## Results and Discussion

### Probe design

In this study we relied for probe design on the species and phylotype description provided by the human oral microbiome database (HOMD) [11], which comprises a collection of 16S rRNA sequences of both cultivable and so far non-cultivable taxa representing the currently known width of bacterial diversity found in the human oral cavity [12]. Oligonucleotide probes were designed with specificity for phylogenetic groups or species of *Lactobacillus, Streptococcus, Lactococcus, Granulicatella *and *Abiotrophia*. Table 1 lists all probes with their sequence and optimum formamide concentration. The latter was determined by systematic optimization in experiments with both reference strains and clinical plaque samples.

**Table 1:** Description and specificity of oligonucleotide probes

Probe	**Target sequence (5' to 3')**^**a**^	Label	**Position**^**b**^	**Target bacteria**^**c**^	**Formamide concentration**^**d**^	Source
LGC358a	CCATTGTGGAAGATTCCCT	Cy3	358-76	Most lactobacilli	25-30	[13], modified
LGC358b-comp	CCATTGCGGAAGATTCCCT	-	358-76	Competitor probe for LGC358a: Staphylococci, *Gemella, Granulicatella, Abiotrophia*	25-30	[13], modified
LAB759	CTACCCATRCTTTCGAGCC	Cy3, FAM	759-77	Most lactobacilli without *L. salivarius *group	30-35	[8]
LAB759-comp	CTACCCACGCTTTCGAGCM	-	759-77	Competitor probe for LAB759: Many streptococci, β-Proteobacteria, but no lactobacilli	30-35	this study
L-Lbre466-2	ACCG**T**CAACCCTT**G**AACAG	Cy3	466-84	*L. brevis*	30-55	this study
L-Lbuc438-2	CACCY**G**TTCTTC**T**CCAACA	FAM	439-57	*L. buchneri *(*L. hilgardii, L. kefiri, L. parabuchneri*)	50-55	this study
Lcas467	CCGTCACGCCGACAACAG	Cy3, FAM	467-84	*L. casei, L. paracasei *subsp*. paracasei, L. rhamnosus, L. zeae*	25-40	this study
L-Lcol732-2	GTTGCAAGC**T**AGACA**G**CC	Cy3	732-49	*L. coleohominis, L. reuteri *(some strains)	≥30	this study
Lfer466	CCGTCAACGTATGAACAG	Cy3	466-83	*L. fermentum*	25	this study
Lfer466-H448	TTACTCTCATACGTGTTC	-	448-65	Helper probe for Lfer466	25	this study
Lfer466-H484	GCCGTGACTTTCTGGTTAAATA	-	484-505	Helper probe for Lfer466	25	this study
Lgas183	GACATGCGTCTAGTGTTG	FAM	183-200	*L. gasserii, L. johnsonii*	25-30	this study
Lgas458	ATAAAGGCCAGTTACTACC	FAM	458-76	*L. acidophilus L. crispatus, L. gasserii, L. jensenii, L. johnsonii (L. amylolyticus, L. amylovorus, L. fornicalis, L. hamsteri, L. helveticus, L. kefiranofaciens, L. kitasatonis)*	25	this study
Lpla759	CTACCCATACTTTCGAGCC	FAM	759-77	*L. paraplantarum, L. plantarum, L. pentosus*	20-30	this study
Lpla990	ATCTCTTAGATTTGCATAGTATG	Cy3	990-1012	*L. paraplantarum, L. plantarum, L. pentosus*	20-35	this study
Lpla990-H1018	CCCGAAGGGAACGTCTA	-	1018-34	Helper probe for Lpla990	20-35	this study
Lreu986	GCGCAAGATGTCAAGACC	Cy3, FAM	986-1004	*L. coleohominis, L. fermentum, L. oris, L. reuterii, L. vaginalis(L. frumenti, L. gastricus, L. ingluviei, L. mucosae, L. panis, L. pontis, L. suebicus)*	25-30	this study
Lreu986-H967	TGGTAAGGTTCTTCGCGTA	-	967-85	Helper probe for Lreu986	25-30	this study
Lsal574	AAAGACCGCCTGCGTTCCC	Cy3, FAM	574-92	*L. salivarius (L. acipiscis, L. animalis, L. apodemi, L. murinus, L. ruminis, L. satsumensis, L. vini)*	35-50	this study
L-Lsal1113-2	CTG**G**CAACT**G**ACAACAAG	FAM	1113-30	*L. salivarius (L. agilis, L. equi, L. saerimneri)*	35-45	this study
Lvag222	ACCGCGGGCCCATCCTGA	Cy3	222-39	*L. vaginalis*	35-50	this study
STR405	TAGCCGTCCCTTTCTGGT	Cy3	405-22	Streptococci	≤ 50	[10,38]
LGC358c	CCATTGCCGAAGATTCCCT	FAM	358-76	Streptococci	25-30	[13], modified
MIT447	CACYCGTTCTTCTCTTACA	FAM	447-65	Mitis group of streptococci	25	[10,38]
MUT590	ACTCCAGACTTTCCTGAC	Cy3	590-607	*Streptococcus mutans*	30	[10,38]
L-Ssob440-2	CACAC**G**TTCTTCCCC**T**AC	FAM	440-57	*Streptococcus sobrinus*	45	this study
L-Sco/int172-2	CAGTAAATGTTCT**T**ATGC**G**GTA	Cy3, FAM	172-93	*Streptococcus constellatus, S. intermedius*	40-55	[39]
ABI161	TGCGGTTTTAGCATCCGT	Cy3	161-78	*Granulicatella adjacens, G. elegans*	≤ 30	this study
ABI1246	AGTTCGCTGCTCGTTGTA	Cy3	1246-63	*Abiotrophia defectiva*, *Granulicatella adjacens, G. elegans, L. coleohominis *(*Facklamia hominis, F. languida, F. miroungae*)	≤ 35	this study
LCC1030	CCTGTATCCCGTGTCCCG	Cy3, FAM	1030-47	*Lactococcus lactis*, *L. garvieae*	40-55	this study
EUB338	GCTGCCTCCCGTAGGAGT	Cy3, FAM	338-55	Most *Eubacteria*	≤ 50	[40]

^a ^Bold printed bases indicate the position of locked-nucleic-acids.

^b ^16S rRNA target position (*Escherichia coli *numbering).

^c ^Taxa in parentheses are detected by the probe but have not been described to colonize the human oral cavity [11].

^d ^Optimum formamide concentration in hybridization buffer.

**Figure 1:**
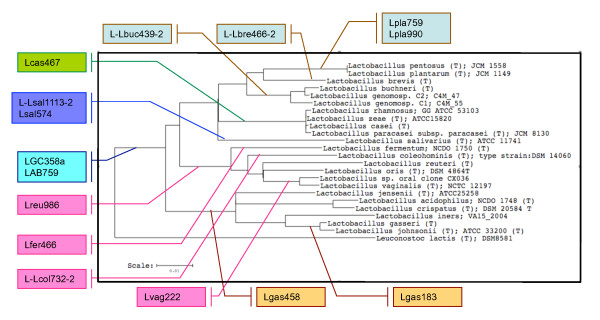
**16S rRNA based phylogenetic tree of oral lactobacilli**. The flags indicate the different oligonucleotide probes used in this study, their colored lines point to the respective phylogenic groups detected by the probes. All listed oral lactobacilli reference strains and phylotypes were retrieved from the Human Oral Microbiome Database [11]. The phylogenetic tree was constructed with *Leuconostoc lactis *as the outgroup using the Tree Builder algorithm of the Ribosomal Data Base Project (http://rdp.cme.msu.edu/index.jsp).

Figure 1 outlines the concept for the design of the probes targeting oral lactobacilli. Two broad *Lactobacillus *probes (LGC358a and LAB759) were generated with the idea that they should complement each other and thus limit the potential of misidentifications [7]. Elongated by one and shifted by four bases LGC358a is a derivative of probe LGC354a [13]. Probes LGC358b (staphylococci and related bacteria) and LGC358c (streptococci) are analogously related to LGC354b and LGC354c described by Meier et al. [13]. As observed often with probes to larger phylogenetic groups, initial experiments with both probes detected besides the targeted lactobacilli significant numbers of false positives (predominantly cocci) when applied to oral plaque samples (see below). *In silico *analyses suggested that these false hybridizations were due to single sequence mismatches and could possibly be avoided by the application of unlabeled competitor probes that are fully complimentary to the targeted 16S rRNA segment of the false positive organisms. Applied in excess together with the labeled FISH probe such competitor probes can increase the differentiation between true- and potential false positives [14]. Thus, LGC358a used in conjunction with LGC358b-comp should recognize selectively most *Lactobacillaceae *organisms and in addition detect parts of the non-oral families *Leuconostocaceae *and *Carnobacteriaceae*, whereas LAB759, when applied together with LAB759-comp (which should suppress recognition of *Streptococcus mutans *as well as *Eikenella, Kingella*, and *Neisseria *sp.) is supposed to identify all oral lactobacilli except *Lactobacillus salivarius *and the majority of *L. fermentum *strains. Application of these competitor probes to various types of plaques samples proved to be successful in providing specificity for lactobacilli (see below). The other probes for lactobacilli were designed to identify bacteria from all major deep branching clusters of the phylogenic tree (Figure 1). Three probes recognize deeply branched, individual species (*L. fermentum, L. salivarius *and *Lactobacillus vaginalis*), which, however, belong to the most frequently detected oral lactobacilli. Probes recognizing oral *Granulicatella *and *Abtiotrophia *species (ABI161 and ABI1246), *Lactococcus lactis *(LCC1030) and cocci belonging to *Streptococcus constellatus/Streptococcus intermedius *(L-Sco/int172-2) or *Streptococcus sobrinus *(L-Ssob440) complement the set of new probes.

### Permeabilization of lactobacilli for FISH

Uniform permeabilization for FISH of fixed lactobacilli (but not of streptococci or *Abiotrophia/Granulicatella*) is a known problem [9], in particular with certain 'notorious' strains. Like other authors before, we have evaluated several permeabilization protocols that precede hybridization and obtained the best results with a modification of a procedure proposed by Harmsen et al. [9] (data not shown). It was applied selectively to all *Lactobacillus *probes and consists of a 5 min exposure to lysozyme and achromopeptidase, followed by a 30 min incubation with lipase.

### Fluorescence intensity and probe specificity

**Figure 2:**
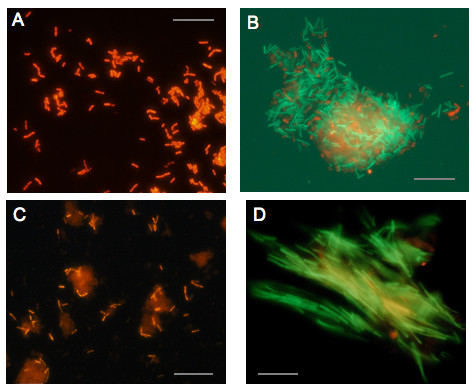
**FISH staining of reference strains and biofilm samples with LAB probes**. (A) *L. rhamnosus *strain AC 413 stained with Lcas467-Cy3 (40% formamide). (B) *L. crispatus *ATCC 33820 stained with both Lfer466-Cy3 (plus the corresponding helper probes) and Lgas458-FAM (25% formamide). The strain should be Lfer466^-^/Lgas458^+^, the FISH assay identified a previously unnoticed contamination with red-stained Lfer466^+ ^cells, which had to be eliminated by recloning. (C) Identification of *L. fermentum *in biofilm 013 using probe Lfer466-Cy3 (plus helper probes; 25% formamide). Note the high proportion of *L. fermentum *in this *in situ *grown biofilm. (D) Sample from the dorsum of the tongue showing an aggregate of large unidentified filaments stained with the *Lactococcus *probe LCC1030-Cy3 and the streptococcal probe L-Sco/int172-2-FAM at 30% formamide. The bacteria are double false positive under these stringency conditions, whereby the detection of the Cy3 fluorescence is hampered by the much stronger FAM fluorescence. To prevent such false positive hybridization, the formamide concentration had to be increased to ≥ 40%. Bars: 10 *μ*m.

*Lactobacillus *probes were tested with 22 reference strains representing the different oral lactobacilli clusters as described by the HOMD (Table 2) and, with the exceptions of probe LAB759 and Lfer466, displayed the anticipated reactivity profile. As an example Figure 2A shows the staining of *Lactobacillus rhamnosus *AC 413 with Lcas467-Cy3. Pointing at one of the strengths of single cell analyses with FISH, strain *Lactobacillus crispatus *ATCC 33820 was found contaminated with *L. fermentum *and required recloning (Figure 2B). With several probes the fluorescence intensity was weak but could be significantly improved by adding non-fluorescent helper probes to the hybridization solution [15], or by employing probes containing locked-nucleic-acids (LNA) [16]. The former bind to regions of the 16S rRNA that are adjacent to the target sequence thereby contributing to the opening of the rRNA's 3-D structure and improving probe accessibility, whereas the latter contain one or two derivative nucleotide analogs with their ribose locked in a C3'-*endo *conformation which leads to a higher target selectivity of the probe. Unexpected from *in silico *data, LAB759 labeled the *L. salivarius *reference strain ATCC 11741 and Lfer466 bound to the *Lactobacillus reuteri *type strain CCUG 33624^T^. The reasons for these exceptional hybridizations remain to be determined. Generally, the LNA-probes yielded high fluorescence intensity but also required high formamide concentrations to display the predicted specificity. In particular, L-Lbre466 was cross-reactive with *Lactobacillus colehominis *and L-Lbuc438 was cross-reactive with some strains of both the *L. casei *and *L. reuteri *clusters if the formamide concentration was kept below 45%. Similarly, the L-Lcol732 probe was false positive with strains from the *Lactobacillus buchneri *cluster at < 40% formamide. Of the other probes listed in Table 1, ABI1246 was strongly positive with all four *Abiotrophia/Granulicatella *reference strains tested (*Granulicatella adjacens *CCUG 27809^T ^and HE-G-R 613A, *Granulicatella elegans *CCUG 38949^T ^and *Abiotrophia defectiva *CCUG 36937), whereas ABI161 labeled only the *Granulicatella *strains. Probe LCC1030 was positive with *Lactococcus lactis *subsp. *lactis *reference strain NCC2211 [17], and the *S. mutans *and *S. sobrinus *probes Smut590 and L-Lsob440 stained reference strains UA159^T ^and OMZ 176, respectively, while none of the probes was positive with strains from other streptococcal species. Probe L-Ssob440-2 yielded better fluorescence intensity than the previously described probe SOB174 [10], but had to be used at high stringency. All these findings were as expected from *in silico *data.

**Table 2:** Reactivity of FISH probes to lactobacilli with target and non-target strains

		16S rRNA probes														
		
Group, Strain	OMZ	LGC358a	LAB759 + LABB759-comp	Lpla759	Lpla990 + H1018	L-Lbre466-2	L-Lbuc438-2	Lcas467	Lsal574	L-Lsal1113-2	Lreu986 + H1018	Lfer466 + H448+ H484	L-Lcol732-2	Lvag222	Lgas458	Lgas183
***L. buchneri *et rel**.																
*L. plantarum *FAM 1638	945	2-4+*^,a^	3-4+	3-4+	2-4+*	-	-	-	-	-	-	-	-	-	-	-
*L. brevis *ATCC 14869	625	3-4 +	2-3 +	-	-	4+	-	-	-	-	-	±	-^b^	-	-	-
*L. brevis *OMZ 1114	1114	2-4+	2-3+*	-	-	3-4+	-	-	-	-	-	-	-^b^	-	-	-
*L. buchneri *ATCC 4005	626	2-4 +	1-2 +	-	-	-	3-4 +	-	-	-	-	-	-^b^	-	-	-
*L. buchneri*	1097	2-4 +*	2-3 +*	-	-	-	3+	-	-	-	-	-	-^b^	-	-	-
***L. casei *et rel**.																
*L. casei *ATCC 393	939	2-4+	3-4+	-	-	-	-^c^	3+	-	-	-	-	-	-	-	-
*L. casei *Cl-16	638	3-4 +	3-4 +	-	-	-	-^c^	3-4 +	-	-	-	-	-	-	-	-
*L. paracasei *ATCC 25598	624	2-4 +*	2-4 +*	-	-	-	-^c^	3-4 +*	-	-	-	-	-	-	-	-
*L. rhamnosus *AC 413	629	2-4 +	2-4 +	-	-	-	-	3-4 +	-	-	-	-	-	-	-	-
*L. rhamnosus *ATCC 7469^T^	602	2-4 +	2-4 +	-	-	-	-	3 +	-	-	-	-	-	-	-	-
***L. salivarius***																
*L. salivarius *ATCC 11741	525	3-4+	3-4+	-	-	-	-	-	2-4+	3-4+	±	-	-	-	-	-
*L. salivarius *OMZ 1115	1115	2-4+	-	-	-	-	-	-	3-4+	3-4+	-	-	-	-	-	-
***L. reuteri *et rel**.																
*L. coleohominis *DSM14060^T^	1113	1-3 +	2-4 +	-	-	-^d^	-	-	-	-	3 +	-	3-4 +	-	-	-
*L. fermentum *ATCC 14931	524	2-4 +*	2 +*^, e^	-	-	-	-	-	-	-	2-4 +	3-4 +	-	-	-	-
*L. fermentum *OMZ 1116	1116	2-4 +	2 +*^, e^	-	-	-	-	-	-	-	2-4 +	3-4 +	-	-	-	-
*L. reuteri *CCUG 33624^T^	1100	2-4 +	3-4 +	-	-	-	-^c^	±	-	-	2-4 +	2-4 +	-	-	-	-
*L. vaginalis *UMCG 5837	1095	2-4 +	3-4 +	-	-	-	-^c^	-	-	-	1-3 +*	-	-	3-4 +	-	-
***L. gasseri *et rel**.																
*L. acidophilus *ATCC 4357	523	2-4+	3-4+	-	-	-	-	-	-	-	±	±	-	-	2-4+	-
*L. crispatus *ATCC 33820	522	3-4 +	3-4 +	-	-	-	-	-	-	-	-		-	-	3-4 +	-
*L. gasseri *ATCC 19992	520	2-4 +	2-4 +	-	-	-	-	-	-	-	±	1 +	-	-	1-3 +	2-4 +
*L. gasseri *OMZ 549	549	2-4 +	2-4 +	-	-	-	-	-	-	-	±	-	-	-	2-4 +	2-4 +
*L. jensenii *UMCG 20557	1096	2-4 +	3-4 +	-	-	-	-	-	-	-	-	-	-	-	-	-

* The asterisk indicates that only a small percentage of the cells could be stained by the probe, in spite of enzymatic pretreatment to improve probe penetration.

^a ^Fluorescence intensity was graded using an arbitrary five-step scale, where - (no fluorescence above background) and 1+ (very faint fluorescence) were considered negative signals, and 2+ (weak), 3+ (strong) and 4+ (brilliant fluorescence) were considered positive signals.

^b ^Probe L-Lcol732-2 labeled *L. brevis *and *L. buchneri *strains at formamide concentrations below 40%.

^c ^L-Lbuc438-2 cross-reacted with certain strains from the *L. casei *and *L. reuteri *groups at formamide concentrations of ≤ 45%.

^d ^L-Lbre466-2 was positive with *L. coleohominis *at ≤ 45% formamide in the hybridization buffer.

^e ^*L. fermentum *was stained with low intensity due to a weak mismatch at position 760.

### Enumeration of lactic acid bacteria from *in situ *formed biofilms

**Figure 3:**
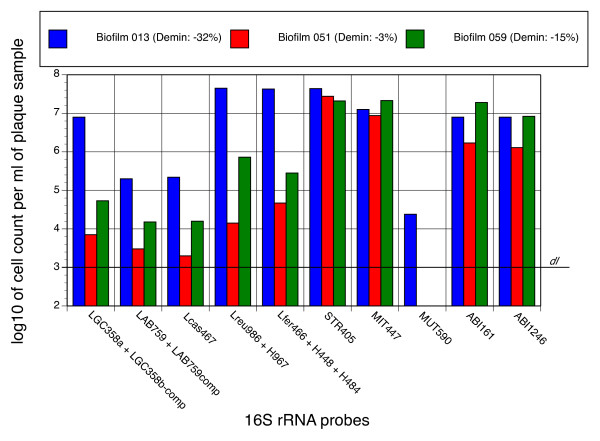
**Enumeration by FISH of lactic acid producing bacteria in three *in situ *grown biofilms**. Biofilms were harvested from bovine enamel discs, carried *in situ *for 10 days and nights by three different volunteers. The discs differed greatly in the extent of demineralization indicated in the within legend of the plot. The detection limit (dl) of the FISH assay was approximately 10^3 ^bacteria per ml of sample.

The applicability of the probes was tested with three *in situ *formed biofilm samples. The samples were harvested from bovine enamel discs carried in acrylic appliances on the buccal side of the mandibular premolar/molar regions [18] by three volunteers whose discs differed greatly in the extent of demineralization (-3%, -15%, and -32%) generated during the 10 days of intermittent extraoral exposure to a 5% glucose/5% sucrose solution. All samples were positive for lactobacilli as detected by the two broadly reactive *Lactobacillus *probes LGC358a and LAB759 (Figure 3). Total cell numbers and numbers of lactobacilli were very similar to findings from an earlier study investigating the microbiota associated with the *in situ *development of caries [19]. Both probes had to be used imperatively together with their respective competitor probes, since, without them, large numbers of cocci and other morphotypes were positive. In the biofilm from disc 013 (biofilm 013 in the following) LGC358a stained clearly two populations of rods that differed in length, whereas LAB759 identified only the shorter of the two morphotypes. The longer and predominant cell type had the probe reactivity profile LGC358a^+^/LAB759^-^/Lfer466^+^/Lreu986^+^/Lcas467^- ^(Figure 2C), whereas the smaller one was LGC358a^+^/LAB759^+^/Lfer466^-^/Lreu986^-^/Lcas467^+^, indicating that the larger rods are *L. fermentum *and the smaller ones lactobacilli from the casei group. While the total number of *L. casei*, streptococci or *Abiotrophia/Granulicatella *seemed not to correlate with the extent of disc demineralization, the high concentration of *L. fermentum *in the biofilm of the extremely demineralized disc 013 was quite remarkable.

All other lactobacillus probes gave negative results. Concerning Lsal574 and Lvag222 we found that both these probes had to be used at much higher stringency conditions (50% formamide) than expected from the *in vitro *experiments with reference strains to prevent cross-reactivity with other biofilm bacteria. In particular cells with the characteristic morphology of *Selenomonas *were often cross-reactive at conditions of insufficient stringency. *Abiotrophia *and *Granulicatella *could be detected in high numbers in all three samples. Both ABI161 and ABI1246 recognized cocci, which in double-labeling experiments stained always negative with the streptococcal probes LGC358c and MIT447 (data not shown). Finally, all samples contained high numbers of streptococci, mostly from the mitis group. *S. mutans*, however, was found with MUT590 in only one sample at low concentration, and the probes for *S. sobrinus *and *S. constellatus*/*S. intermedius *gave negative results. Identification by FISH of streptococci, in particular of the mitis group, is hindered severely by high conservation of the 16S rRNA gene sequence among these taxa [20,21] and therefore FISH detection of oral streptococci still relies mostly on phylogenic group-specific probes. A surprise finding, confirmed with supragingival plaque samples and scrapings from the dorsum of the tongue, was that both *Lactococcus *probe LCC1030 and *S. constellatus/intermedius *probe L-Sco/int172-2 triggered rather strong fluorescence of long filaments with blunted ends (Figure 2D), which could only be suppressed by applying formamide concentrations exceeding 40%. The results were confirmed when probes with exchanged fluorescence labels were used (Cy3 instead of 6-FAM and vice versa). Potential specificity problems associated with phylogenetic group-specific probes are a known phenomenon [7,22]. However, screening of the RDP10 database for oral bacteria with this type of morphology and ≤ 2 sequence mismatches within the gene fragments complementary to these probes, failed to reveal any hints about the possible identity of these filaments. Experiments aiming at their isolation by fluorescence activated cell sorting are ongoing.

### Typing of *Lactobacillus *isolates from *in situ *grown oral biofilms

With the aim to verify the identification by FISH of the lactobacilli present in the three *in situ *grown biofilm samples (Figure 3), aliquots were cultured on LBS agar. Five strains (OMZ 1117-1121), representing the various colony types observed, were isolated and characterized by both FISH and partial sequencing of the 16S rDNA (Table 3). Sequence analysis identified two strains as *L. fermentum *(OMZ 1117 and 1121) [EMBL: FR667951] and two as *L. casei/L. paracasei *(OMZ 1118 and 1120) [EMBL: FR667952], based on 100% sequence similarity with respective reference strains. The fifth strain was typed as *L. vaginalis *(OMZ 1119) [EMBL: FR667953] with a sequence match score of 0.995 to reference strain Dox G3. *L. vaginalis *had not been detected by direct FISH analysis of the biofilms (Figure 3), presumably because the cell number was below the detection limit of approximately 10^3 ^bacteria per ml of sample suspension. Tested by FISH with the whole set of probes all five isolates showed the anticipated profile (Table 3). The two *L. fermentum *isolates were negative to weakly positive with LAB759 in repeated experiments. This is explained by *L. fermentum *strains having an adenine at position 760 of their 16S rRNA, as opposed to a cytosine at the corresponding position of probe LAB759. This peripheral mismatch may result sometimes in weak cross-reactivity (see also *L. fermentum *strains in Table 2). In summary, typing by gene sequencing corroborated the data obtained from the direct FISH analysis of the *in situ *grown biofilms.

**Table 3:** Identification and FISH reactivity profiles of five isolates from *in situ *biofilms 013, 051 and 059

	Isolated strain (biofilm of origin)				
	
	OMZ 1117 (013)	OMZ 1118 (013)	OMZ 1119 (051)	OMZ 1120 (051)	OMZ 1121 (059)
***16S rRNA probes***					
LGC358a	2-4 +	3-4 +	3-4 +	3-4 +	3 +
LAB759 + LAB759-comp	- to 2 +	3-4 +	3-4 +	3 +	- to 2 +
Lpla759	-	-	-	-	-
Lpla990+ H1018	-	-	-	-	-
L-Lbre466	-	-	-	-	-
L-Lbuc438	-	- ^a^	- ^a^	- ^a^	-
Lcas467	-	4 +	-	3-4 +	-
Lsal574	-	-	-	-	-
L-Lsal1113	-	-	-	-	-
Lreu986 + H967	2-4 +	-	3-4 +	-	3-4 +
Lfer466 + H448 + H484	2-4 +	-	-	-	3-4 +
L-Lcol732	-	-	-	-	-
Lvag222	-	-	3-4 +	-	-
Lgas458	-	-	-	-	-
Lgas183	-	-	-	-	-
***Identification***^**c**^	*L. fermentum*	*L. paracasei L.casei*	*L. vaginalis*	*L. paracasei L.casei*	*L. fermentum*

^a ^Positive at ≤ 45% formamide.

^b ^Scoring of fluorescence intensity is described in a footnote to Table 2.

^c ^Species identification was based on ≥ 99.5% 16S rRNA gene sequence similarity with reference strains listed in RDP10 (http://rdp.cme.msu.edu/index.jsp). The respective partial 16S rRNA gene sequences of OMZ 1117 and 1121 [EMBL: FR667951], and of OMZ 1118 and 1120 [EMBL: FR667952] were identical. OMZ 1119 was identified as *L. vaginalis *[EMBL: FR667953].

### Critical importance of several assay parameters

Lactobacilli are difficult targets for FISH because of their cell wall's resistance to probe penetration. The protocol used successfully in the present study to increase cell permeability evolved from the method of Harmsen *et al. *[9], which we supplemented with achromopeptidase, previously described to open cell walls of *Actinomyces *strains [23,24]. Systematic evaluation of this three-enzyme-pretreatment with 12 reference strains from seven *Lactobacillus *species showed its indispensability. However, a minority of strains proved to be particularly resistant, as up to 20% of the cells recognizable by phase contrast could not be stained. Of course this raised concerns that such false-negative results could also affect analyses of clinical samples. We cannot completely rule out this possibility, but after comprehensive analysis of many plaque samples we would like to hypothesize that there are differences in cell wall permeability between cultured and native lactobacilli and that false-negative cells are primarily seen after FISH with cultured lactobacilli.

With cell wall permeability remaining a potential reason for concern, maximum fluorescence intensity from penetrated probes is essential. Fluorescence intensity depends on cellular ribosome content, *in situ *probe accessibility to the probe target region, and rRNA stability [25]. Several procedures to maximize the performance of FISH probes have been described [15,16,26,27]. They alter the 3-dimensional structure of the target region by using helper probes, optimize probe length and hybridization conditions, improve binding affinity by modifying the probes' backbone with LNA substitutions, or inhibit enzymatic rRNA degradation. In this study we used all four procedures to improve fluorescence intensity of certain probes. For Lfer466, Lreu986, and Lpla990 one or two helper probes binding directly adjacent to the target site were added to the hybridization solution and in each case a clear-cut improvement of fluorescence intensity was observed. The same was the case when the LNA-substituted probe L-Ssob440-2 was compared to Ssob440. For five other probes the decision to opt for LNA insertions was taken solely based on own and published experience [25], suggesting limited accessibility of the probes' target site. All these LNA/DNA-probes displayed intensive fluorescence, but required strict adherence of very stringent hybridization conditions for sufficient specificity.

## Conclusions

In this study we have described the application of 20 new phylogenetic group- or species-specific oligonucleotide probes for the single-cell detection of oral LAB in various clinical or experimental biofilms. The results show that the probes can be used for the rapid identification and quantification of a broad range of LAB for both research and diagnostic purposes, provided that restrictions concerning cell permeabilization (lactobacilli), hybridization stringency, fluorescence intensity optimization, and use of competitor probes are considered. Although designed to cover the diversity of oral lactobacilli, these probes should prove of value far beyond the field of oral microbiology, as many of them detect non-oral species and phylogenetic groups of importance to gastroenterology, gynecology, heart diseases, food industry, etc. Gene sequence typing of isolated strains confirmed the results obtained by analyzing biofilm samples directly by FISH. On a speculative note, the apparent correlation between the *L. fermentum *cell number and the extent of demineralization seen with the three samples from the *in situ *study could indicate that these bacteria have played a significant role in the carious process. The abundance of *L. fermentum *might be explained by high resistance to low pH giving these bacteria a selective ecological advantage during the formation of the biofilm.

## Methods

### Strains, plaque samples and *in situ *grown biofilms

*Lactobacillus *reference strains (listed in Table 2) were grown in 10% CO_2 _at 37 °C on LBS (*Lactobacillus *selection) agar and in LBS broth (Becton Dickinson). *Lactococcus, Streptococcus, Abiotrophia and Granulicatella *reference strains from the OMZ strain collection were propagated anaerobically on Columbia blood agar or in fluid universal medium [28]. They were harvested after 24-36 h during the late log-phase of growth. Supragingival plaque samples and scrapings from the dorsum of the tongue were collected from two of the authors, washed in 0.9% NaCl, fixed in 4% paraformaldehyde/PBS (20 min, 4 °C), and stored in 50% ethanol at -20 °C.

*In situ *grown biofilm samples were harvested from bovine enamel discs (6.8 mm Ø) carried for 10 days and nights by three volunteers in the course of a double-blind split-mouth de- and remineralization study carried out at the University of Bergen, Bergen, Norway [18]. The Regional Committee for Medical Research Ethics Western Norway approved the study protocol and the volunteers gave their informed written consent to participate in the study. Inclusion criteria for volunteers were normal salivary flow and a full dentition without non-restored caries lesions or evidence of moderate or severe gingivitis. Antibiotics, mouth rinses or tooth pastes containing antimicrobial agents (*e.g. *chlorhexidine, triclosan, SnF_2_, Zn^2+^, etc.) or drugs affecting the salivary flow rate should not have been used for the last three months. The appliances were kept in 0.9% NaCl during meals and tooth cleaning; in addition they were dipped seven times daily for 10 min in 5% glucose/5% sucrose solution to promote plaque formation. After 10 days in the oral cavity of the volunteers, the discs were removed from the appliances and the biofilms were harvested by scraping with plastic scalers (ZI 10; Deppeler, Rolle, Switzerland) into approximately 1 ml of sterile 0.9% NaCl as collecting fluid (exact volume determined for each sample). The samples were frozen at -80 °C and shipped to Zürich on dry ice for further analyses. There, freshly defrosted samples were vortexted for 1 min, sonicated for 5 s, aliquoted and assessed by FISH. Aliquots were also grown at 37 °C anaerobically and in 10% CO_2 _on LBS agar (Becton Dickinson) with the aim to isolate and type representative strains by partial 16S rDNA sequencing. Demineralization of discs was determined by quantitative light-induced fluorescence as described [29].

### Preparation of multi-well slides for FISH

Overnight cultures of lactobacilli (LBS broth) were washed in 0.9% NaCl, diluted in coating buffer [30], spotted on 18- or 24-well slides (Cel-Line Associates), air-dried, and fixed in 4% paraformaldehyde/PBS (20 min, 4 °C). Analogously, *in situ *grown biofilm samples, supragingival plaque samples and tongue scrapings were vortexed at maximum speed for 60 s, diluted in coating buffer and coated to 18- or 24-well slides as described [30]. To improve cell wall permeability each well selected for FISH of lactobacilli was treated individually at room temperature first for 5 min with 9 *μl *of lysozyme (1 mg ml^-1^; Sigma-Aldrich L-7651) and achromopeptidase (1 mg ml^-1^; Sigma-Aldrich A-7550) in Tris-HCl (pH 7.5) with 5 mM EDTA, and then for 30 min with 9 *μ*l of lipase (Sigma-Aldrich L-1754; at 25 mg ml^-1 ^in water the lipase suspension was centrifuged for 5 min at 16'000 × *g *after which the supernatant was used). Thereafter, to limit unspecific FISH probe binding all wells were covered for 30 min at 37 °C with 9 *μ*l of PBS containing Denhardt's solution (Fluka 30915; diluted 1:50) in the presence of protectRNA RNase inhibitor (Sigma-Aldrich R-7397; diluted 1:500) [15,16,26,27]. At the end of the respective incubation periods the solutions were carefully aspirated and the slides briefly washed in wash-buffer (0.9% NaCl, 0.05% Tween 20, 0.01% NaN_3_), dipped in water, and air-dried. All solutions were made with water of nano-pure quality.

### Fluorescent *in situ *hybridization

The 16S rRNA targeted oligonucleotide probes used in this study are listed in Table 1. Custom-synthesized by Microsynth, they were labeled at 5'-end with Cy3 or 6-FAM, or in some cases at both ends with 6-FAM. Probes marked by "L-" in front of the probe name, contain one or two LNA to improve *in situ *hybridization efficiency [16]. Probes were designed as described previously [30] using the ARB software [31] with the SILVA rRNA database [32,33] and additional rRNA sequence information from 'The Ribosomal Data Base Project II' [34,35] and the 'National Center for Biotechnology Information' [36]. Probes LGC358a and LAB759 had to be used in conjunction with a competitor probe to suppress cross-reactivity with taxa other than lactobacilli and few probes were used together with helper probes [15] to improve fluorescence intensity (Table 1). Probes were used at final concentrations of 5 ng *μ*l^-1 ^(Cy3 conjugates) or 15 ng *μ*l^-1 ^(FAM conjugates, competitor and helper probes). EUB338 served as positive control. FISH was performed as described [30] using hybridization times of 2 or 4 h and probe-specific formamide concentrations as listed in Table 1. Optimum formamide concentrations were determined by varying the formamide concentrations systematically between 25% and 55% in FISH experiments with both reference strains and oral biofilm samples.

### Scoring and enumeration of stained bacteria

Following FISH, air-dried multiwell slides were covered with mounting fluid (90% glycerol in PBS with 25 mg g^-1 ^1,4-diazabicyclo[2,2,2]octan) and cover-slips. Bacteria stained by FISH were enumerated as described using an Olympus BX60 epifluorescence microscope (Olympus Optical [Schweiz]) [30]. Scoring of fluorescence intensity is described in a footnote to Table 2.

### 16S rDNA sequencing

Partial 16S rRNA gene sequences of five lactobacillus isolates (OMZ 1117-1121) from the three *in situ *grown biofilms were determined as described previously [35]. The sequences of 1393, 1360, 1366, 1371 and 1379 bp in length were compared to gene bank data of the The Ribosomal Data Base Project using the Seq Match algorithm [33]. Identification of isolates was based on ≥ 99.5% similarity. The sequences of OMZ 1117 - 1119 were deposited at EMBL with accession numbers FR667951 - FR667953.

## Authors' contributions

BQ and HLS carried out and read FISH analyses. VZ and TT contributed to probe design and editing of the manuscript. EG and BG designed and carried out the *in situ *study and participated in editing the manuscript. RG designed the project and the probes, analyzed FISH experiments and wrote the manuscript. All authors read and approved the final manuscript. The authors declare no conflict of interest.

## References

[B1] Aas JA Paster BJ Stokes LN Olsen I Dewhirst FE Defining the normal bacterial flora of the oral cavity J Clin Microbiol 2005 43 5721 5732 10.1128/JCM.43.11.5721-5732.2005 16272510 PMC1287824

[B2] Kilian M Borriello P, Murray PR, Funke G *Streptococcus *and *Lactobacillus* Topley and Wilson's Microbiology and Microbial Infections 2005 London: Hodder Arnold 833 881

[B3] Marsh PD Martin MV Oral Microbiology 2009 5 Edinburgh: Churchill Livingstone Elsevier

[B4] Marsh PD Nyvad B Fejerskov O, Kidd E. Chichester The oral microflora and biofilms on teeth Dental Caries: The Disease and Its Clinical Management 2008 2 UK: Wiley-Blackwell 163 187

[B5] Baddour LM Virulence factors among gram-positive bacteria in experimental endocarditis Infect Immun 1994 62 2143 2148 8188334 10.1128/iai.62.6.2143-2148.1994 PMC186490

[B6] Husni RN Gordon SM Washington JA Longworth DL *Lactobacillus *bacterimia and endocarditis: Review of 45 cases Clin Infect Dis 1997 25 1048 1055 10.1086/516109 9402355

[B7] Amann R Fuchs BM Single-cell identification in microbial communities by improved fluorescence *in situ *hybridization techniques Nat Rev Micro 2008 6 339 348 10.1038/nrmicro1888 18414500

[B8] Zijnge V van Leeuwen MBM Degener JE Abbas F Thurnheer T Gmür R Harmsen HJM Oral biofilm architecture on natural teeth PLoS ONE 2010 5 e9321 10.1371/journal.pone.0009321 20195365 PMC2827546

[B9] Harmsen HJM Elfferich P Schut F Welling GW A 16S rRNA-targeted probe for detection of lactobacilli and enterococci in faecal samples by fluorescent *in situ *hybridization Microb Ecol Health Dis 1999 11 3 12 10.1080/089106099435862

[B10] Thurnheer T Gmür R Giertsen E Guggenheim B Automated fluorescent *in situ *hybridization for the specific detection and quantification of oral streptococci in dental plaque J Microbiol Methods 2001 44 39 47 10.1016/S0167-7012(00)00226-8 11166098

[B11] The human oral microbiome database http://www.HOMD.org

[B12] Chen T Yu WH Izard J Baranova OV Lakshmanan A Dewhirst FE The Human Oral Microbiome Database: a web accessible resource for investigating oral microbe taxonomic and genomic information Database (Oxford) 2010 2010 baq013 20624719 10.1093/database/baq013 PMC2911848

[B13] Meier H Amann R Ludwig W Schleifer KH Specific oligonucleotide probes for *in situ *detection of a major group of gram-positive bacteria with low DNA G+C content System Appl Microbiol 1999 22 186 196 10.1016/S0723-2020(99)80065-4 10390869

[B14] Manz W Amann R Ludwig W Wagner M Schleifer KH Phylogenetic oligonucleotide probes for the major subclasses of proteobacteria: problems and solutions System Appl Microbiol 1992 15 593 600

[B15] Fuchs BM Glöckner FO Wulf J Amann R Unlabeled helper oligonucleotides increase the *in situ *accessibility to 16S rRNA of fluorescently labeled oligonucleotide probes Appl Environ Microbiol 2000 66 3603 3607 10.1128/AEM.66.8.3603-3607.2000 10919826 PMC92190

[B16] Kubota K Ohashi A Imachi H Harada H Improved *in situ *hybridization efficiency with locked-nucleic-acid-incorporated DNA probes Appl Environ Microbiol 2006 72 5311 5317 10.1128/AEM.03039-05 16885281 PMC1538721

[B17] Comelli EM Guggenheim B Stingele F Neeser JR Selection of dairy bacterial strains as probiotics for oral health Eur J Oral Sci 2002 110 218 224 10.1034/j.1600-0447.2002.21216.x 12120707

[B18] Giertsen E Guggenheim B Thurnheer T Gmür R Microbiological aspects of an *in situ *model to study effects of antimicrobial agents on dental plaque ecology Eur J Oral Sci 2000 108 403 411 10.1034/j.1600-0722.2000.108005403.x 11037756

[B19] Thomas RZ van der Mei HC van der Veen MH de Soet JJ Huysmans MCDNJM Bacterial composition and red fluorescence of plaque in relation to primary and secondary caries next to composite: an in situ study Oral Microbiol Immunol 2008 23 7 13 10.1111/j.1399-302X.2007.00381.x 18173792

[B20] Kawamura Y Whiley RA Shu SE Ezaki T Hardie JM Genetic approaches to the identification of the mitis group within the genus *Streptococcus* Microbiology 1999 145 2605 2613 10517614 10.1099/00221287-145-9-2605

[B21] Kilian M Poulsen K Blomqvist T Håvarstein LS Bek-Thomsen M Tettelin H Sørensen UBS Evolution of *Streptococcus pneumoniae *and its close commensal relatives PLoS ONE 2008 3 e2683 10.1371/journal.pone.0002683 18628950 PMC2444020

[B22] Barr JJ Blackall LL Philip B Further limitations of phylogenetic group-specific probes used for detection of bacteria in environmental samples ISME J 2010 4 1 3 10.1038/ismej.2010.37 20357832

[B23] Bowden G Johnson J Schachtele C Characterization of *Actinomyces *with genomic DNA fingerprints and rRNA gene probes J Dent Res 1993 72 1171 1179 10.1177/00220345930720080201 8360358

[B24] Sekar R Pernthaler A Pernthaler J Warnecke F Posch T Amann R An improved protocol for quantification of freshwater *Actinobacteria *by fluorescence *in situ *hybridization Appl Environ Microbiol 2003 69 2928 2935 10.1128/AEM.69.5.2928-2935.2003 12732568 PMC154517

[B25] Behrens S Rühland C Inacio J Huber H Fonseca A Spencer-Martins I Fuchs BM Amann R *In situ *accessibility of small-subunit rRNA of members of the domains *Bacteria, Archaea, *and *Eucarya *to Cy3-labeled oligonucleotide probes Appl Environ Microbiol 2003 69 1748 1758 10.1128/AEM.69.3.1748-1758.2003 12620867 PMC150112

[B26] Yilmaz LS Okten HE Noguera DR Making all parts of the 16S rRNA of *Escherichia **coli *accessible *in situ *to single DNA oligonucleotides Appl Environ Microbiol 2006 72 733 744 10.1128/AEM.72.1.733-744.2006 16391113 PMC1352245

[B27] Gmür R Lüthi-Schaller H A combined immunofluorescence and fluorescent *in situ *hybridization assay for single cell analyses of dental plaque microorganisms J Microbiol Methods 2007 69 402 405 17258338 10.1016/j.mimet.2006.12.012

[B28] Gmür R Guggenheim B Antigenic heterogeneity of *Bacteroides intermedius *as recognized by monoclonal antibodies Infect Immun 1983 42 459 470 6196291 10.1128/iai.42.2.459-470.1983 PMC264452

[B29] Gmür R Giertsen E van der Veen MH de Josselin de Jong E ten Cate JM Guggenheim B *In vitro *quantitative light-induced fluorescence to measure changes in enamel mineralization Clin Oral Invest 2006 10 187 195 10.1007/s00784-006-0058-z 16810532

[B30] Züger J Lüthi-Schaller H Gmür R Uncultivated *Tannerella *BU045 and BU063 are slim segmented filamentous rods of high prevalence but low abundance in inflammatory disease-associated dental plaques Microbiology 2007 153 3817 3829 17975090 10.1099/mic.0.2007/010926-0

[B31] Ludwig W Strunk O Westram R Richter L Meier H Yadhukumar Buchner A Lai T Steppi S Jobb G Förster W Brettske I Gerber S Ginhart AW Gross O Grumann S Hermann S Jost R König A Lüßmann R May M Nonhoff B Reichel B Strehlow R Stamatakis AP Stuckmann N Vilbig A Lenke M Ludwig T Bode A Schleifer KH ARB: a software environment for sequence data Nucleic Acids Res 2004 32 1363 1371 10.1093/nar/gkh293 14985472 PMC390282

[B32] Pruesse E Quast C Knittel K Fuchs BM Ludwig W Peplies J Glöckner FO SILVA: a comprehensive online resource for quality checked and aligned ribosomal RNA sequence data compatible with ARB Nucl Acids Res 2007 gkm864. 17947321 10.1093/nar/gkm864 PMC2175337

[B33] Silva - Comprehensive Ribosomal RNA Database http://www.arb-silva.de/

[B34] Cole JR Chai B Farris RJ Wang Q Kulam SA McGarrell DM Garrity GM Tiedje JM The Ribosomal Database Project (RDP-II): sequences and tools for high-throughput rRNA analysis Nucleic Acids Res 2005 33 D294 D296 10.1093/nar/gki038 15608200 PMC539992

[B35] Ribosomal Database Project http://rdp.cme.msu.edu

[B36] Basic Local Alignment Search Tool (BLAST) http://blast.ncbi.nlm.nih.gov/Blast.cgi 10.1101/pdb.top17 21357135

[B37] Gmür R Munson MA Wade WG Genotypic and phenotypic characterization of fusobacteria from Chinese and European patients with inflammatory periodontal diseases Syst Appl Microbiol 2006 29 120 130 16464693 10.1016/j.syapm.2005.07.011

[B38] Paster BJ Bartoszyk IM Dewhirst FE Identification of oral streptococci using PCR-based, reverese-capture, checkerboard hybridization Meth Cell Sci 1998 20 223 231 10.1023/A:1009715710555

[B39] Guggenheim B Gmür R Galicia JC Stathopoulou P Benakanakere MR Meier A Thurnheer T Kinane D *In vitro *modeling of host-parasite interactions: the 'subgingival' biofilm challenge of primary human epithelial cells BMC Microbiol 2009 9 280 10.1186/1471-2180-9-280 20043840 PMC2818713

[B40] Amann RI Binder BJ Olson RJ Chisholm SW Devereux R Stahl DA Combination of 16S rRNA-targeted oligonucleotide probes with flow cytometry for analyzing mixed microbial populations Appl Environ Microbiol 1990 56 1919 1925 2200342 10.1128/aem.56.6.1919-1925.1990 PMC184531

